# Differential Response of Coral Assemblages to Thermal Stress Underscores the Complexity in Predicting Bleaching Susceptibility

**DOI:** 10.1371/journal.pone.0159755

**Published:** 2016-07-20

**Authors:** Loke Ming Chou, Tai Chong Toh, Kok Ben Toh, Chin Soon Lionel Ng, Patrick Cabaitan, Karenne Tun, Eugene Goh, Lutfi Afiq-Rosli, Daisuke Taira, Rosa Celia Poquita Du, Hai Xin Loke, Aizat Khalis, Jinghan Li, Tiancheng Song

**Affiliations:** 1 Tropical Marine Science Institute, National University of Singapore, 18 Kent Ridge Road, Singapore, 119227; 2 National Biodiversity Centre, National Parks Board, 1 Cluny Road, Singapore, 259569; 3 DHI Water and Environment, 1 Cleantech Loop, 03-04 CleanTech One, Singapore, 637141; 4 Department of Biological Sciences, National University of Singapore, 14 Science Drive 4, Singapore, 117543; 5 Maritime and Port Authority of Singapore, 460 Alexandra Road PSA Building, Singapore, 119963; Biodiversity Research Center, Academia Sinica, TAIWAN

## Abstract

Coral bleaching events have been predicted to occur more frequently in the coming decades with global warming. The susceptibility of corals to bleaching during thermal stress episodes is dependent on many factors and an understanding of these underlying drivers is crucial for conservation management. In 2013, a mild bleaching episode ensued in response to elevated sea temperature on the sediment-burdened reefs in Singapore. Surveys of seven sites highlighted variable bleaching susceptibility among coral genera–*Pachyseris* and *Podabacia* were the most impacted (31% of colonies of both genera bleached). The most susceptible genera such as *Acropora* and *Pocillopora*, which were expected to bleach, did not. Susceptibility varied between less than 6% and more than 11% of the corals bleached, at four and three sites respectively. Analysis of four of the most bleached genera revealed that a statistical model that included a combination of the factors (genus, colony size and site) provided a better explanation of the observed bleaching patterns than any single factor alone. This underscored the complexity in predicting the coral susceptibility to future thermal stress events and the importance of monitoring coral bleaching episodes to facilitate more effective management of coral reefs under climate change.

## Introduction

Thermal stress events that cause disturbances to natural ecosystems are predicted to occur every ten to twenty years [1,2]. Prolonged elevation of sea temperatures exerts tremendous stress on coral reefs, and scleractinian corals are especially susceptible to such impacts. Photo-inhibition and the subsequent expulsion of symbiotic algae in response to sustained temperature elevation lead to coral bleaching [3]. Consequently, the reduction of photosynthetic activity creates an energy deficit which cannot be fully compensated by heterotrophic feeding alone [4]. This disruption of the coral-zooxanthellae symbiotic relationship has been linked to large-scale coral mortality [5]. Major bleaching episodes can decimate up to 70% of the corals within three months of the onset of bleaching and can impact hundreds of kilometers of reefs [5].

Not all coral genera bleach to the same extent during thermal stress events and susceptibility variation can play an important role in shaping the resultant community structure and species diversity of a reef [6]. The different responses are determined by a range of intrinsic factors, such as taxon, growth form, and colony size. For example, Marshall and Baird (2000) [6] reported that acroporids and pocilloporids are the most susceptible to thermal stress, while corals of the genera *Cyphastrea*, *Turbinaria* and *Galaxea* are among the most resistant. Branching corals are also more prone to bleaching due to reduced coral tissue thickness and morphology-dependent mass transfer of heat and metabolites [7]. In addition, smaller coral colonies withstood thermal stress better due to the higher mass transfer rates as compared to larger colonies [8].

The variability in bleaching prevalence is also influenced by a myriad of environmental factors, including the magnitude of thermal stress and irradiance [9,10], efficiency of water circulation for heat dissipation [11,12] and the thermal stress history of the locality [13]. While some generalizations on coral bleaching susceptibility have been made [6, 7], the interactions among these factors are potentially more complex than currently assumed. For instance, observations of the trends in bleaching susceptibility during the 2010 mass bleaching event suggested that fast-growing branching corals may not be as vulnerable to thermal stress as is commonly perceived [14].

There is a growing body of work documenting coral community responses to bleaching in recent years [13,14,15]. However, most appear to have focused on major bleaching events where over 50% of the colonies had been impacted [6,14,16]. Punctuating the major episodes are minor bleaching events where effects are localized, with less than 25% bleaching observed on the reef [16,17]. Unlike major bleaching events, corals that bleach during minor episodes tend to be those which are especially vulnerable to heat stress [16]. Yet, there is little research on the response of coral assemblages during these minor events [16,17]. Additionally, with the effects of future mass bleaching episodes likely exacerbated by multiple stressors [2,15], it is pertinent to examine how reefs under chronic disturbance by human activities will fare under warming sea surface temperature. There is a need to examine coral bleaching episodes at a much finer scale, to enhance our understanding of the mechanisms underlying coral susceptibility to thermal stress.

Singapore’s reefs persist in an environment which has been impacted by intensive coastal development and land reclamation for over five decades [18]. Today, they are dominated by foliose and massive corals that are tolerant to high sedimentation and low light conditions [18,19,20]. Contemporary sedimentation rates can be as high as 20 mg cm^-2^ day^-1^ [20], but the reefs continue to support a rich diversity of corals [21]. They were not spared from widespread bleaching-related coral mortality caused by sustained elevated sea surface temperatures in 1998 and 2010 [14,22]. More recently, between June to July 2013, sea surface temperature (SST) in Singapore (30.6°C) [23] exceeded the maximum monthly mean (29.8°C) [23], and an average of 6% bleaching at multiple reefs was recorded (this study). The number of bleached colonies was less than 25%, thus the bleaching episode was considered minor [16]. In the present study, we surveyed the southern offshore reefs to examine if there were spatial variations in the bleaching patterns and if there were differential responses to bleaching among coral genera.

## Materials and Methods

### Study sites

**Fig 1:**
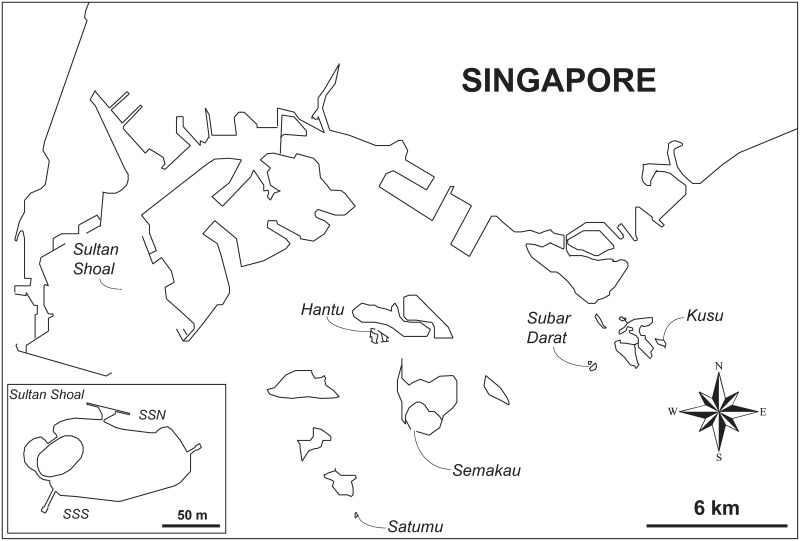
Map of the southern islands of Singapore, with inset showing Sultan Shoal. The seven study sites are indicated (SSN = Sultan Shoal North; SSS = Sultan Shoal South).

Surveys were carried out at seven sites fringing the offshore islands south of mainland Singapore—Hantu (01°13.645′N, 103°44.780′E), Semakau (01°11.51'N, 103°45.32′E), Kusu (1°13.32’N, 103°51.33’E), Subar Darat (1°12.54’N, 103°49.53’E), Satumu (1°9.36’N, 103°44.24’E), Sultan Shoal North (1°14.23’N, 103°38.55’E) and South (1°14.21’N, 103°38.52’E) (Fig 1). A research permit for this work was granted by the Singapore government through the National Parks Board.

### Coral bleaching surveys

The surveys were conducted from May to July 2013 and coincided with the ocean warming event that occurred from June to July 2013 (30.6°C) [23]. At each site, surveys were carried out by establishing six 20-m transects at a depth of 2 to 7 m on the reef. Along each replicate transect, photographs of ten 1m x 1m quadrats (each spaced 1m apart) were taken (total of 60 quadrats per reef). All hard corals within the quadrats were enumerated and identified to genus initially following Veron, 2000 [24]. In line with recent developments in scleractinian taxonomy, the genus names were updated following Wallace et al. 2012 [25] for *Acropora*; Benzoni et al. 2010 [26] for *Psammocora*; Budd et al. 2012 [27], Huang et al. 2014 [28] for Merulinidae, Lobophylliidae and Diploastraeidae. For Fungiidae, the classification followed Gittenberger et al. 2011 [29] and Hoeksema 2009 [30] except for the solitary free-living members such as *Fungia* (Veron, 2000 [24]). These were categorised as “other solitary fungiids” as voucher specimens were not collected for identification to reflect the latest taxonomic name changes. For all other taxa that were yet to be revised, identification followed Veron, 2000 [24].

For each colony, the proportion of the total area that bleached was classified as two levels of severity: (1) 1–50% and (2) 51–100% following Guest *et al*. (2012) [14]. Coral bleaching susceptibility, defined as the percentage of colonies that bleached relative to the total number of colonies of a particular genus, was then calculated for each transect [31]. The maximum diameters of the bleached colonies were measured using the software CPCe (Coral Point Count with Excel Extension).

### Factors driving the variation in bleaching response

To examine the drivers of differential bleaching response, three factors–coral genus, site, and size (maximum colony diameter) of the bleached corals were examined. Data from four coral genera with the most number of bleached colonies (*Pachyseris*, *Dipsastraea*, *Pectinia* and *Porites*) were analysed and the corresponding bleaching responses (bleached and non-bleached) were modelled using generalized linear model calculated with binary logistic regression in R 2.14.2. Model selection was done using the Akaike Information Criterion (AIC). Models with lower AIC values were selected which corresponded to the final model that best explained the data.

## Results

### Coral bleaching response

**Fig 2:**
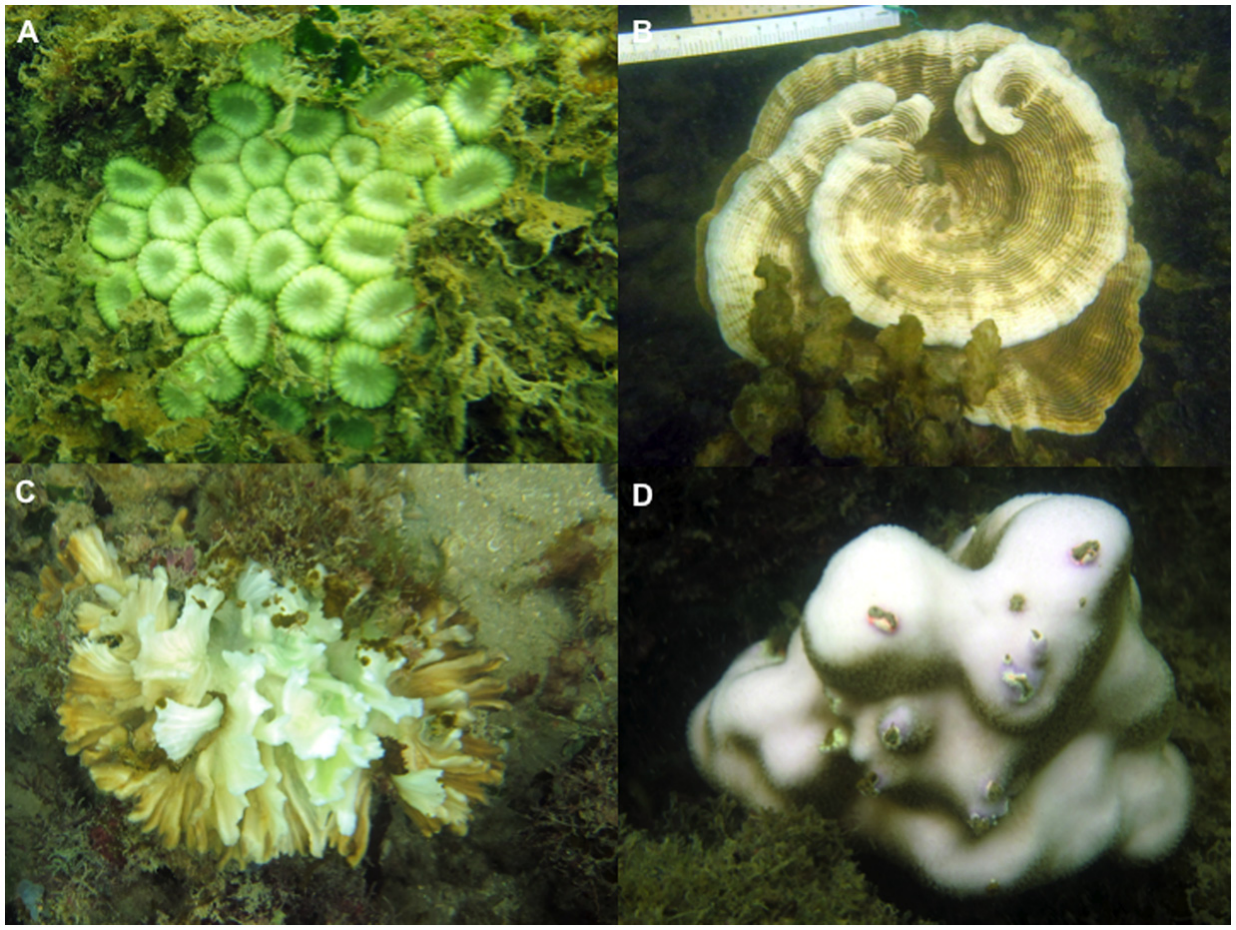
Coral genera that had the most number of bleached colonies during the 2013 minor bleaching episode in Singapore. Coral genera included (a) *Dipsastraea*, (b) *Pachyseris*, (c) *Pectinia* and (d) *Porites*.

A total of 2648 colonies were observed. As 6.1% (162) of the colonies were bleached, this qualified as a minor episode. Twenty-three of the 37 coral genera showed signs of bleaching (Table 1, Fig 2). Among the genera, *Pachyseris* and *Podabacia* had the highest bleaching susceptibility of 31% and 30.8% respectively and collectively accounted for 39.5% (64) of the total number of bleached colonies. The remaining bleached colonies (60.5%) were distributed across the other 21 genera, which had bleaching susceptibilities of between 1.2% and 16.7%. Fourteen genera did not bleach during this episode, including *Acropora* and *Pocillopora*. Bleaching severity was evenly distributed between Levels 1 (<50% bleached) and 2 (>50% bleached), with 54.3% of the corals in the former and 45.7% in the latter categories.

**Table 1:** Bleaching response of coral genera from seven study sites during a minor bleaching episode in Singapore in 2013.

Genera	*n*	Number of bleached colonies	Bleaching susceptibility (%)	Number of corals in Level 1 (<50% bleached)	Number of corals in Level 2 (>50% bleached)
*Pachyseris*	200	62	31.0	37	25
*Podabacia*	13	4	30.8	2	2
*Lithophyllon*	18	3	16.7	1	2
*Goniastrea*	7	1	14.3	1	0
*Platygyra*	85	9	10.6	6	3
*Porites*	105	10	9.5	5	5
*Diploastrea*	12	1	8.3	1	0
*Goniopora*	69	5	7.2	2	3
*Pectinia*	421	27	6.4	15	12
*Coscinaraea*	33	2	6.1	1	1
*Lobophyllia*	19	1	5.3	1	0
*Dipsastraea*	215	10	4.7	3	7
*Pavona*	72	3	4.2	2	1
*Symphyllia*	48	2	4.2	1	1
*Hydnophora*	24	1	4.2	1	0
*Turbinaria*	104	3	2.9	3	0
*Montipora*	117	3	2.6	1	2
Other soliary fungiids	90	2	2.2	0	2
*Merulina*	290	6	2.1	3	3
*Astreopora*	55	1	1.8	0	1
*Echinopora*	61	1	1.6	0	1
*Leptoria*	65	1	1.5	1	0
*Favites*	324	4	1.2	1	3
*Alveopora*	50	0	0	-	-
*Galaxea*	23	0	0	-	-
*Cyphastrea*	22	0	0	-	-
*Ctenactis*	21	0	0	-	-
*Oxypora*	20	0	0	-	-
*Mycedium*	17	0	0	-	-
*Acropora*	11	0	0	-	-
*Oulastrea*	10	0	0	-	-
*Pocillopora*	10	0	0	-	-
*Herpolitha*	6	0	0	-	-
*Acantastrea*	5	0	0	-	-
*Psammocora*	2	0	0	-	-
*Plerogyra*	2	0	0	-	-
*Euphyllia*	2	0	0	-	-
**Total**	**2648**	**162**	**6.1**	**88**	**74**

Of all the sites surveyed in 2013, corals at Sultan Shoal North, Hantu and Subar Darat had the highest coral bleaching susceptibilities of 12.4%, 11.7% and 11.5% respectively (Table 2). The number of bleached colonies (98) recorded from these sites accounted for more than 60% of the total bleached coral count while the remaining sites had bleaching susceptibilities of between 2.3% to 6.0%. Although Sultan Shoal South is located just 200 m from Sultan Shoal North on the same island, the former was one of the sites with corals of the lowest susceptibility (2.5%).

**Table 2:** Bleaching response of coral colonies from seven study sites during a minor bleaching episode in Singapore in 2013.

Site	*n*	Number of bleached colonies	Bleaching susceptibility (%)	Number of corals in Level 1 (<50% bleached)	Number of corals in Level 2 (>50% bleached)
Sultan Shoal North	242	30	12.4	11	19
Hantu	349	41	11.7	25	16
Subar Darat	235	27	11.5	23	4
Kusu	317	19	6	12	7
Semakau	474	20	4.2	7	13
Sultan Shoal South	476	12	2.5	6	6
Satumu	555	13	2.3	8	5
Total	2648	162	6.1	88	74

**Fig 3:**
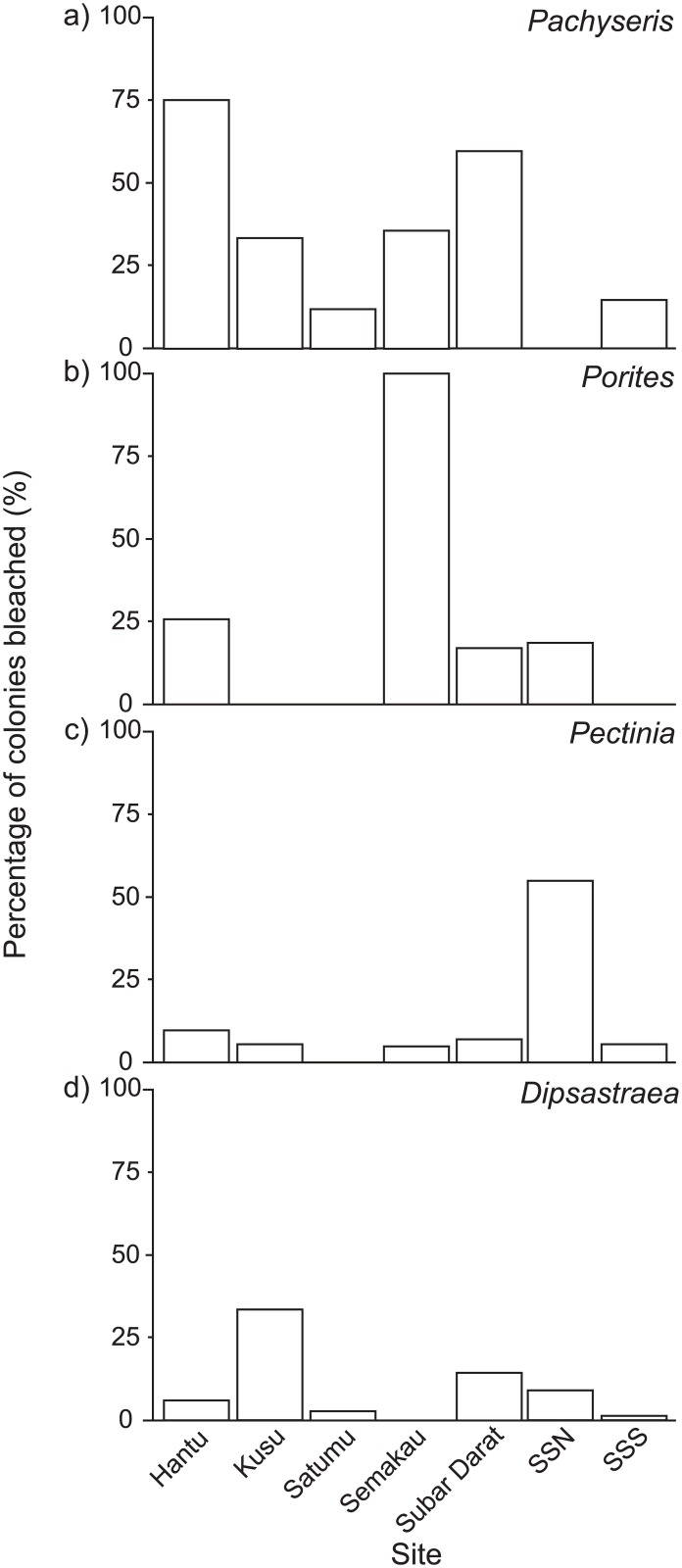
Coral bleaching susceptibility across seven study sites during a minor bleaching episode in Singapore in 2013. Four genera with the largest number of bleached colonies in each site are presented: (a) *Pachyseries*, (b) *Porites*, (c) *Pectinia* and (d) *Dipsastraea*. Corals of the genus *Pachyseris* were not recorded at Sultan Shoal North (SSS = Sultan Shoal South; SSN = Sultan Shoal North).

The order of susceptibility among taxa varied across sites and this was clearly observed in the bleaching responses of the four genera with the largest number of bleached colonies (Fig 3). At Sultan Shoal North where bleaching susceptibility was the highest, *Pectinia* was the most bleached, followed by *Porites* and *Dipsastraea*. However, the order was reversed at Hantu (i.e. *Pachyseris*, *Dipsastraea*, *Porites* and *Pectinia*) and Subar Darat (i.e. *Pachyseris*, *Porites*, *Dipsastraea* and *Pectinia)*.

### Drivers of differential bleaching response

All the single-factor models (either size, site or genus) performed significantly better (*p <* 0.05) than the null model and each model explained up to 19% of the total variation observed (Table 3). This indicated that all three factors were driving the bleaching patterns observed in this study. Among these single-factor models, size was the best predictor (R^2^ = 0.19), with larger colonies (29.44 ± 23.74 cm) more likely to bleach than the smaller ones (14.24 ± 9.35 cm) (Table 4). Among the four genera (see Table 1), *Pachyseris* was the most susceptible to bleaching (31%), followed by *Porites* (9.5%), *Pectinia* (6.4%) and *Dipsastraea* (4.7%). Of the seven sites (see S1 Table), the corals at Subar Darat (24.6%), Hantu (24.6%), Sultan Shoal North (18%) and Kusu (18%) were the most susceptible to bleaching, while Semakau (11%), Satumu (7.9%) and Sultan Shoal South (4.3%) were the least affected.

**Table 3:** Performances of logistic regression models using the independent variables: site, size and genera, to account for the observed bleaching responses.

Model^a^	AIC	R^2^
Null	704.7	0
Site	653.8	0.12
Size	607.7	0.19
Genus	616.3	0.18
Site + Size	540.8	0.33
Site + Genus	550.2	0.32
Size + Genus	580.0	0.25
Site + Size + Genus	493.8	0.41
Site + Size + Genus + Site:Size + Site:Genus + Size:Genus^b^	483.0	0.51

^a^All the models and the corresponding variables were significant at *p =* 0.05 confidence level. Interaction between variables is denoted by a colon.

^b^ Best model was chosen using the Akaike Information Criterion (AIC)

**Table 4:** Mean maximum diameter (± SD) of the corals from the four genera with the most number of bleached colonies.

Genus	Unbleached colonies (cm)	Bleached colonies (cm)	Overall (cm)
*Dipsastraea*	11.76 ± 6.72	16.00 ± 5.68	11.95 ± 6.73
*Pachyseris*	21.33 ± 14.26	37.87 ± 27.61	26.95 ± 21.27
*Pectinia*	12.82 ± 7.18	17.04 ± 6.24	13.09 ± 7.19
*Porites*	15.69 ± 8.73	20.00 ± 8.37	16.14 ± 8.75
Overall (cm)	14.24 ± 9.35	29.44 ± 23.74	16.12 ± 13.06

The multiple-factor models performed much better than single-factor ones—the best dual-factor model and tri-factor model accounted for up to 33% and 41% of the total variation respectively (Table 3). Models with a combination of extrinsic factor (site) and intrinsic factors (genus or size) were better than the model with only intrinsic factors (Table 3).

When interactions among all three factors were considered, the best model that took into account all possible pairwise interactions accounted for 10% more variation than the best non-interaction model (Table 3). Apart from the interaction between size and site, other interactions contributed significantly (*p <* 0.05) to the model.

## Discussion

Severe thermal stress leading to major bleaching events has been linked to large-scale coral mortality [5] and a rapid loss of reef ecosystem function [2]. Thermal stress events that are milder can also cause bleaching across reefs, albeit on a smaller scale [16]. Even though the elevation in sea surface temperatures in Singapore between June and July 2013 was lower than the bleaching threshold temperature (31°C) [23], coral bleaching was observed at numerous offshore reefs, and the affected coral colonies surveyed exhibited varying degrees of bleaching at all sites. The current study demonstrated that even during minor episodes, bleaching response can vary with coral genus, site and colony size. More importantly, the findings underscore the immense complexity in predicting coral bleaching responses.

Our results deviate from general perceptions of the susceptibility of coral genera to thermally-induced bleaching. Genera such as *Pocillopora* and *Acropora* have been widely deemed as the most susceptible to thermal stress, as were observed from previous bleaching events across the world [6,32,33]. However, none of the *Acropora* and *Pocillopora* colonies bleached in the present study. Instead, massive corals from the genera *Goniastrea*, *Platygyra* and *Porites* which are usually moderately susceptible to thermal stress [7,14], were among the most affected during this bleaching episode. This atypical trend in bleaching susceptibility was similarly observed from the 2010 major bleaching event in Singapore and Peninsular Malaysia [14]. Thermal stress events, such as those in 1998 and 2010, might exert tremendous selection pressure on coral populations by eliminating thermally susceptible colonies and facilitating the propagation of tolerant ones [13]. Since genera such as *Acropora* grow fast and achieve sexual maturity early, they can adapt rapidly to environmental change [34].

As the coral assemblages among sites were not dissimilar, the spatial variations in scleractinian diversity were unlikely to have influenced the differences in bleaching responses, unlike those observed from other studies [6]. Instead, the differences observed in this study indicate that extrinsic factors are crucial drivers of site-specific bleaching patterns. Corals at sites such as Satumu and Semakau were less affected by thermal stress than others, even though all study sites are at most 23 km apart and hence relatively near to each other. The most striking difference was observed at Sultan Shoal, where the northern reef had the highest bleaching susceptibility of all sites (12%) while the southern reef was one of the least affected (2.5%). From *in situ* measurements obtained in 2014, the 2013 bleaching patterns at some sites appeared to be driven by water flow. High water motion can dissipate heat along the colony surfaces faster and was reported to be effective in reducing thermal stress to corals [11]. Similar to a previous study (Taira *et al*., In review), the reef at Kusu was consistently exposed to higher water motion and had lower bleaching susceptibility than those at Sultan Shoal. The results however indicate that it is insufficient to attribute water flow as the only abiotic driver in bleaching response. For instance, the reef at Hantu was subjected to faster water flow than Sultan Shoal North, but both sites were similarly affected by bleaching, while both sites at Sultan Shoal had registered similar sedimentation rates and turbidity but elicited different bleaching responses (Unpublished data).

It is thus evident that the myriad factors driving coral bleaching responses cannot be adequately addressed independently. For instance, although the larger colonies were more affected by bleaching, as was also reported from other studies [7,8], colony size only accounted for 19% of the total variation in this study. In addition, the order of genus-specific bleaching susceptibility differed substantially among sites. For example, the order of bleaching susceptibility at Sultan Shoal North was radically different from Sultan Shoal South, Hantu and Subar Darat. Eventually, the regression model that fit best was one that incorporated size, genus and site, as well as the corresponding interactions in the analyses. Such interactions are not unexpected, as there have been observations that corals in deeper reefs were less susceptible to bleaching [6]. Our results highlight the generalization of current perceptions of bleaching susceptibility, as it is apparent that corals can respond differently when the various factors are examined in concert.

The present study underscores the importance of re-evaluating the conventional paradigm of “winners” and “losers” during bleaching events. Coral genera (e.g. *Pachyseris* and *Podabacia*) that may have been less impacted by thermal stress during previous bleaching events in Singapore were instead most affected during the 2013 minor bleaching episode. In sharp contrast, coral genera generally perceived as most vulnerable (e.g. *Acropora* and *Pocillopora*), fared better. Clearly, it is essential to monitor coral assemblages during both minor and major bleaching episodes to provide a more comprehensive evaluation of bleaching response in an era of climate change [35,36]. However, factors such as coral taxa or site [7,14] which are used commonly as predictors of bleaching susceptibility appear to gloss over other critical drivers of bleaching response, while other considerations (e.g. depth, water flow, sedimentation) are usually not examined in detail. The paucity of factors investigated thus impedes the coherent and systematic understanding of coral bleaching responses [37] and highlights the inadequacy of current monitoring methods. While there are resource constraints in reef monitoring [38,39], our findings demonstrate that all three factors examined in this study (genus, site and size) are important in augmenting the bleaching response prediction model and we strongly recommend that these factors be incorporated as part of future bleaching monitoring efforts.

## Supporting Information

S1 Table Bleaching response among sites and genera. Total number of corals recorded in all seven sites and the corresponding bleaching prevalence for each genera (SS = Sultan Shoal).
(PDF)
