# Supplementary material for: Differential Response of Coral Assemblages to Thermal Stress Underscores the Complexity in Predicting Bleaching Susceptibility
Source: PLoS One. 2016 Jul 20;11(7):e0159755. doi: 10.1371/journal.pone.0159755 (PMC4954682; doi:10.1371/journal.pone.0159755)
Supplement: S1 Table — Total number of corals recorded in all seven sites and the corresponding bleaching prevalence for each genera (SS = Sultan Shoal). (PDF) [file pone.0159755.s001.pdf]

S1 Table. Bleaching response among sites and genera. Total number of corals recorded in all seven sites and the corresponding bleaching prevalence for each genera (SS = Sultan Shoal).

	**Hantu**		**Kusu**		**Satumu**		**Semakau**		**Subar Darat**		**SS North**		**SS South**		**Total**	
Coral genera	***n***	**%**	***n***	**%**	***n***	**%**	***n***	**%**	***n***	**%**	***n***	**%**	***n***	**%**	***n***	**%**
Pachyseris	35	57	24	33	83	12	31	35	20	60	0	-	7	14	200	31
Podabacia	0	-	1	0	1	100	2	0	0	-	2	100	7	0	13	30.8
Lithophyllon	0	-	0	-	6	0	0	-	1	0	3	100	8	0	18	16.7
Goniastrea	0	-	3	0	2	0	1	100	0	-	0	-	1	0	7	14.3
Platygyra	4	50	16	6	9	0	11	0	3	67	24	17	18	0	85	10.6
Porites	20	25	21	0	20	0	2	100	12	17	11	9	19	0	105	9.5
Diploastrea	0	-	1	0	6	0	3	0	0	-	0	-	2	50	12	8.3
Goniopora	26	15	8	0	3	0	24	0	2	50	0	-	6	0	69	7.2
Pectinia	53	9	20	5	54	0	118	4	45	7	11	55	120	6	421	6.4
Coscinaraea	0	-	15	13	5	0	0	-	2	0	10	0	1	0	33	6.1
Lobophyllia	0	-	0	-	0	-	14	0	5	20	0	-	0	-	19	5.3
Dipsastraea	17	6	6	33	39	3	36	0	14	14	34	9	69	1	215	4.7
Pavona	13	0	29	0	15	0	1	0	4	75	4	0	6	0	72	4.2
Symphyllia	3	33	5	0	8	0	8	0	5	0	6	17	13	0	48	4.2
Hydnophora	4	0	2	50	4	0	10	0	1	0	2	0	1	0	24	4.2
Turbinaria	29	0	12	17	17	0	10	0	10	0	12	8	14	0	104	2.9
Montipora	5	20	26	4	60	0	3	0	8	0	6	0	9	11	117	2.6
Solitary fungiids	5	0	1	0	17	0	32	0	12	0	3	33	20	5	90	2.2
Merulina	55	4	33	0	43	2	86	1	11	0	22	9	40	0	290	2.1
Astreopora	3	0	6	17	6	0	8	0	6	0	16	0	10	0	55	1.8
Echinopora	2	0	15	0	12	0	5	0	10	0	1	100	16	0	61	1.6
Leptoria	8	0	11	0	10	0	11	0	8	13	9	0	8	0	65	1.5
Favites	33	0	27	0	94	0	30	0	31	0	50	8	59	0	324	1.2
Alveopora	27	0	0	-	4	0	3	0	12	0	2	0	2	0	50	0
Galaxea	2	0	3	0	6	0	2	0	1	0	5	0	4	0	23	0
Cyphastrea	0	-	16	0	0	-	0	-	1	0	4	0	1	0	22	0
Ctenactis	1	0	0	-	5	0	8	0	0	-	0	-	7	0	21	0
Oxypora	1	0	1	0	10	0	7	0	0	-	1	0	0	-	20	0
Mycedium	2	0	2	0	3	0	4	0	3	0	0	-	3	0	17	0
Acropora	0	-	1	0	10	0	0	-	0	-	0	-	0	-	11	0
Oulastrea	0	-	3	0	0	-	1	0	1	0	3	0	2	0	10	0
Pocillopora	0	-	5	0	3	0	0	-	2	0	0	-	0	-	10	0
Herpolitha	0	-	0	-	0	-	1	0	5	0	0	-	0	-	6	0
Acanthastrea	0	-	3	0	0	-	1	0	0	-	0	-	1	0	5	0
Plerogyra	1	0	0	-	0	-	0	-	0	-	1	0	0	-	2	0
Psammocora	0	-	1	0	0	-	1	0	0	-	0	-	0	-	2	0
Euphyllia	0	-	0	-	0	-	0	-	0	-	0	-	2	0	2	0
**Total**	349	11.7	317	6.0	555	2.3	474	4.2	235	11.5	242	12.4	476	2.5	2648	6.1
